# Surgical resection of brain and adrenal gland metastases from gastric cancer: a case report and literature review

**DOI:** 10.1093/jscr/rjae163

**Published:** 2024-03-21

**Authors:** Yuto Kitano, Shigekazu Ohyama, Yasumichi Yagi, Ichiro Onishi, Masato Kayahara

**Affiliations:** Department of Surgery, National Hospital Organization Kanazawa Medical Center, Kanazawa 920-8650, Japan; Department of Surgery, National Hospital Organization Kanazawa Medical Center, Kanazawa 920-8650, Japan; Department of Surgery, National Hospital Organization Kanazawa Medical Center, Kanazawa 920-8650, Japan; Department of Surgery, National Hospital Organization Kanazawa Medical Center, Kanazawa 920-8650, Japan; Department of Surgery, National Hospital Organization Kanazawa Medical Center, Kanazawa 920-8650, Japan

**Keywords:** gastric cancer, brain metastasis, long-term survival

## Abstract

The prognosis of recurrent gastric cancer is generally poor, and aggressive surgical treatment is rarely performed. Herein, we present the case of a patient who underwent resection of cerebellar and adrenal gland metastases from gastric cancer. The patient was treated for gastric cancer with distal gastrectomy at 23 years and for remnant gastric cancer with completion gastrectomy at 48 years. At 59 years old, she experienced vertigo and nausea and was diagnosed with cerebellar and left adrenal gland tumours. First, the cerebellar tumours were resected and diagnosed as metastases of gastric cancer. After 1 month, the adrenal gland tumour was resected and diagnosed as metastatic. She underwent whole-brain radiotherapy and subsequent chemotherapy with S-1. One year after the surgery, the patient died of meningitis carcinomatosa. There are few reports on long-term survival after the resection of brain metastases. Herein, we report our experience along with a review of the literature.

## Introduction

The prognosis of recurrent gastric cancer is generally poor. Chemotherapy is the standard treatment for recurrent gastric cancer, and surgery is rarely indicated, except in cases of local recurrence or liver metastasis [1]. Metastases from gastric cancer usually affect the lymph nodes, peritoneum, liver, lungs, and bones. The frequency of brain metastases has been reported to be <1% in patients with gastric cancer [2, 3]. Brain metastases have often progressed at the time of detection, and surgical treatment is rarely performed. We present a case of metastatic recurrence in the brain and adrenal glands after gastrectomy for advanced gastric cancer.

## Case report

**Figure 1:**
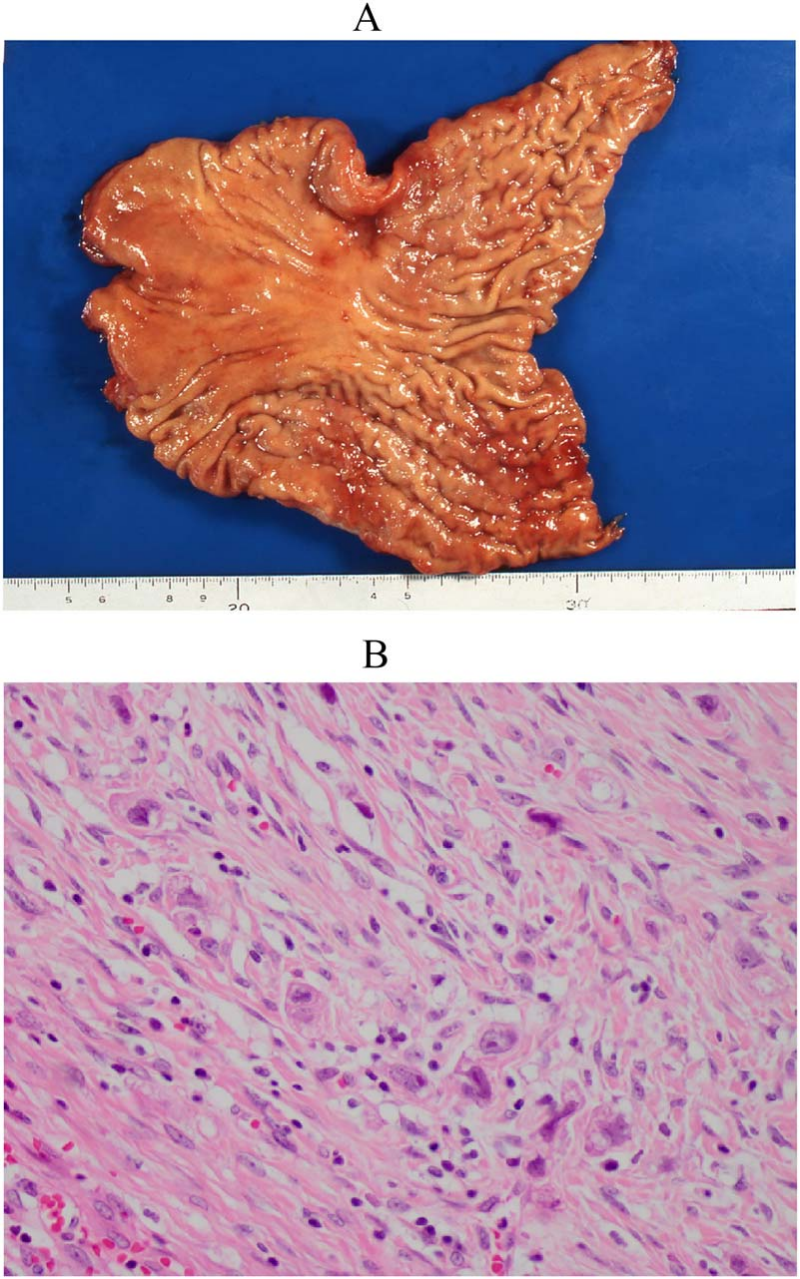
Resected specimen of the first gastrectomy. (A) A type 4 tumour was detected at the anterior of the angular lesion. (B) Poorly differentiated adenocarcinoma was detected (HE, ×400).

**Figure 2:**
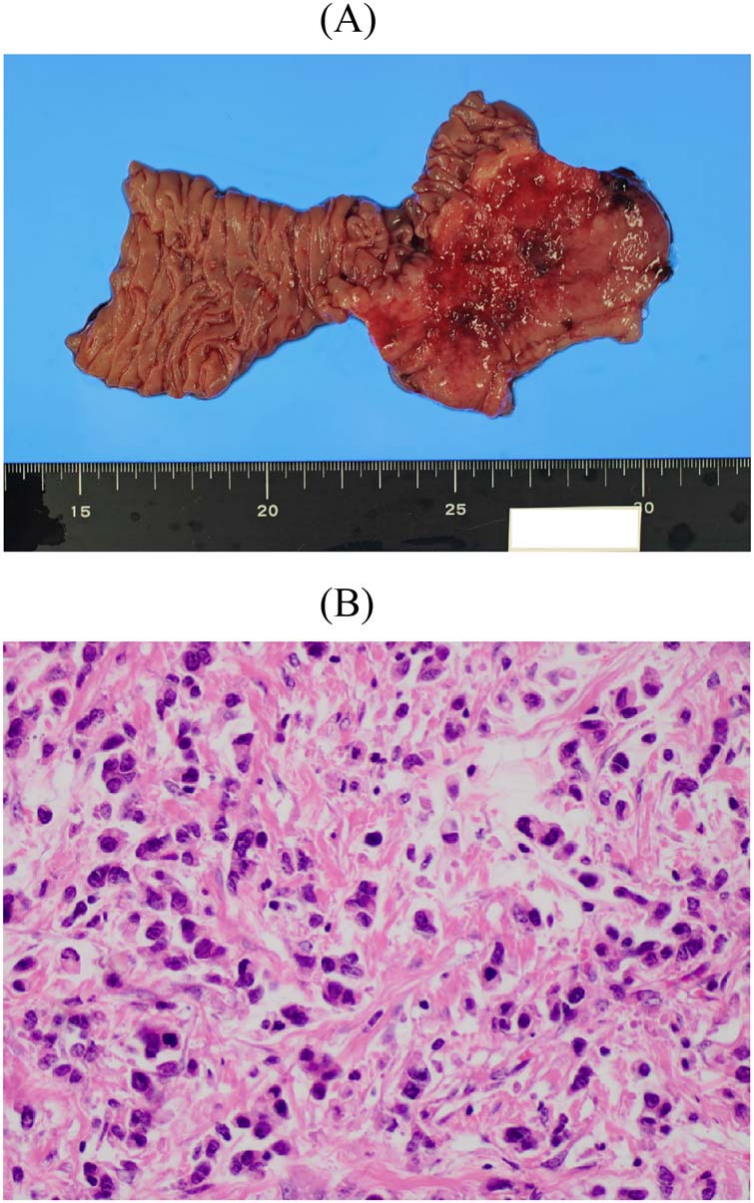
Resected specimen of the second gastrectomy. (A) A type 4 tumour was detected at the entire remnant stomach. The surgical margins were negative. (B) Poorly differentiated adenocarcinoma (non-solid type) was detected (HE, ×400).

A female patient underwent subtotal gastrectomy for advanced gastric cancer at 23 years. Histopathological findings indicated poorly differentiated carcinoma, type4, se, ly1, v0, and six lymph node metastases (Fig. 1). Her peritoneal lavage cytology results were negative. Adjuvant chemotherapy with mitomycin C was initiated. At 48 years (after 25 years), she underwent a complete gastrectomy for remnant gastric cancer. Histopathological findings indicated signet-ring cell carcinoma, type4, se, ly2, v3, and four lymph node metastases (Fig. 2). The peritoneal lavage cytology results were negative. Adjuvant chemotherapy with S-1 was administered for 2 years. At 59 years (11 years after secondary gastrectomy), she experienced vertigo and nausea. She was then referred to our hospital.

**Figure 3:**
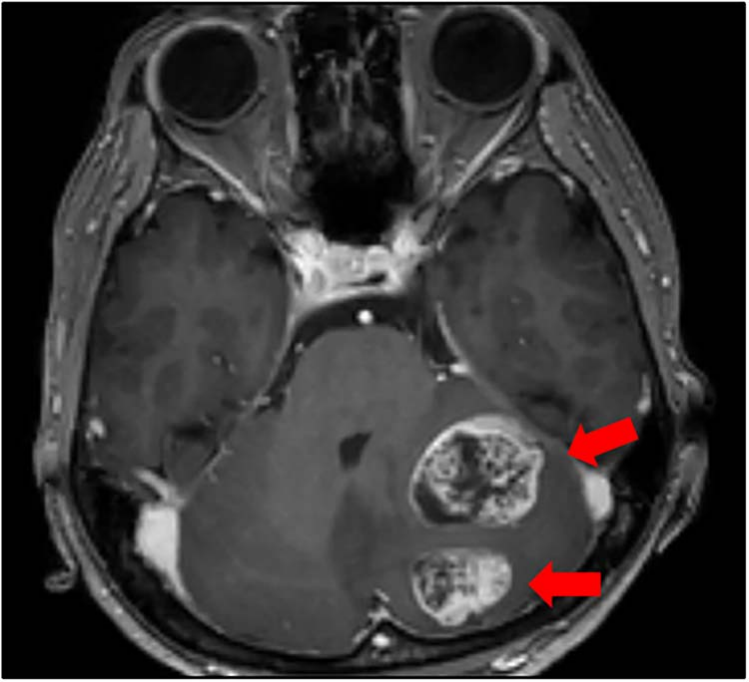
Cranial MRI findings (T1-weighted image). Two ring-enhanced masses with maximum diameters of 3.3 and 2.4 cm were observed in the left cerebellum.

**Figure 4:**
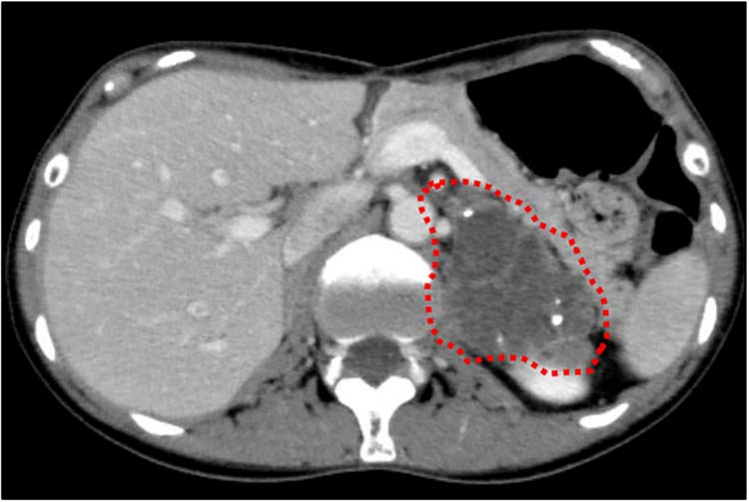
Abdominal CT findings. A calcified tumour with a maximum diameter of 6.0 cm was observed in the left adrenal gland.

**Figure 5:**
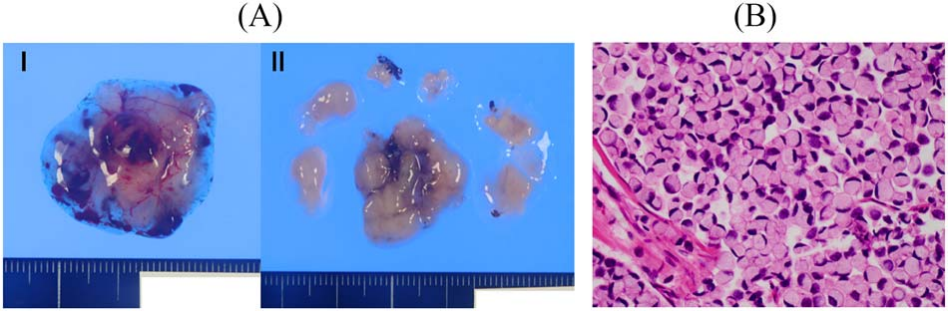
Resected specimen of the cerebellar tumours. (A) Two resected specimens of the cerebellum. (B) Signet-ring cell carcinoma was detected (HE, ×400).

Cranial magnetic resonance imaging (MRI) showed two nearby ring-shaped enhanced tumours with maximum diameters of 3.3 and 2.4 cm in the left cerebellum (Fig. 3). Contrast-enhanced computed tomography (CT) revealed swelling of the left adrenal gland with a maximum diameter of 6 cm (Fig. 4). Positron emission tomography showed other high fluorine-18-deoxyglucose accumulations without the above lesions. The tumour markers, including CEA, CA19-9, and CA125, were within normal ranges. The cerebellar tumours were resected and diagnosed because the patient had neurological symptoms. Histopathological findings of the resected specimens indicated signet-ring cell carcinoma (Fig. 5), which was diagnosed as metastatic gastric cancer. Resection of intracranial tumours rapidly improved her neurological disturbances.

**Figure 6:**
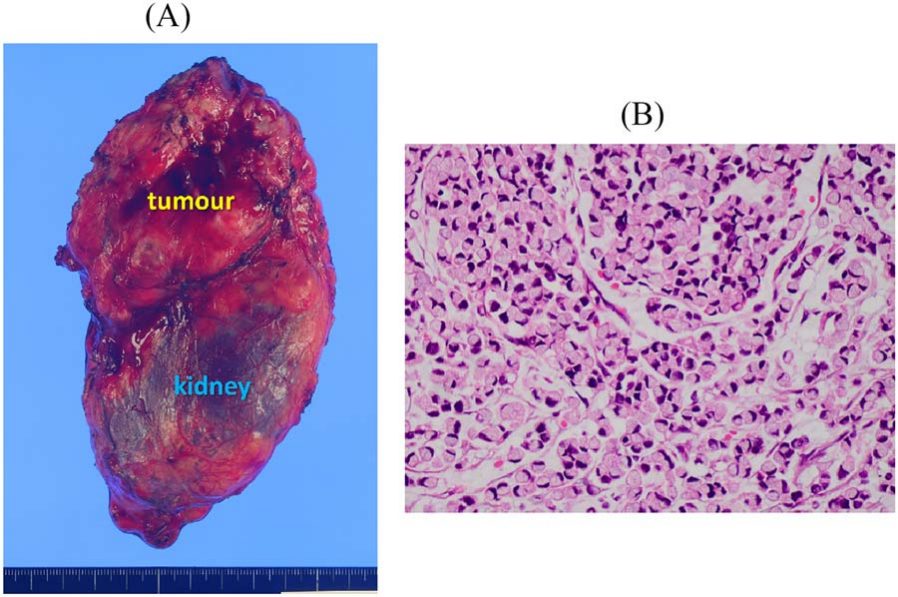
Resected specimen of the left adrenal gland tumour. (A) The tumour resected with the left kidney. (B) Signet-ring cell carcinoma was detected (HE, ×400).

After 1 month, the left adrenal gland tumour was resected via adrenonephrectomy. The tumour did not invade the diaphragm or other surrounding organs and was marginally resected. The histopathological findings indicated signet-ring cell carcinoma (Fig. 6). She underwent whole brain radiotherapy (WBRT) and subsequent chemotherapy with S-1 after metastatic tumour resection. Ten months after the last operation, she developed meningitis carcinomatosa and died 2 months later.

## Discussion

Brain metastases from gastrointestinal cancer are infrequent, and central nervous system metastases are rarely examined during staging. Imaging studies may be performed when a patient experiences central nervous symptoms. Moreover, it has been reported that the frequency of brain metastasis is 0.47–0.7% in patients with gastric cancer [2, 3]. In patients with gastric cancer accompanied by brain metastasis, concurrent systemic metastases are often present and mostly involve bone (46%), liver (42%), and lung (29%) metastases [2, 3]. Few cases have been reported in which recurrent brain metastatic lesions were resected following the removal of primary gastric cancer.

A PubMed search yielded 10 cases of surgical resection of recurrent brain metastases from gastric or esophagogastric junction (EGJ) cancers published between 2000 and 2024 (Table 1) [3–9]. Herein, we summarize 11 surgical cases, including our case. They comprised seven men and four women with a median age of 71 (53–76) years. The primary sites of the cancers were located in the EGJ or U lesions of the stomach in eight patients. Seven patients underwent radiotherapy after intracranial surgery, and six underwent chemotherapy. The median interval between the initial operation and the detection of brain metastasis was 24 (4–132) months. The interval in our case was the longest compared with those reported in the literature. The median survival period after brain metastasis was 7 (1–96) months.

**Table 1:** Summary of patients with brain metastases from gastric cancer treated by surgical resection

No.	Author	Report year	Age	Sex	Primary site (location, depth, histology)	Lymph node metastasis	Interval between initial operation and brain metastasis (months)	Numerous of brain metastasses	Treatment for brain metastasis	Survival after brain metastasis (months)
1	Kasakura *et al.* [3]	2000	74	M	ND/ND/tub1	ND	18	Solitary	Surgery, RT	7
2		2000	59	M	U/ss/por	N(+)	12	Solitary	Surgery, CT	3
3		2000	53	M	ND/ND/tub1	ND	4	Solitary	Surgery, CT	6
4	Tawada *et al.* [4]	2014	76	M	U/ss/tub2	N(−)	24	Solitary	Surgery, SRS, CT	5
5	Matsunaga *et al.* [5]	2014	71	M	EGJ/ss/tub2	N(+)	72	Solitary	Surgery, SRS, CT	66
6		2014	64	M	EGJ/ss/pap	N(+)	48	Solitary	Surgery, SRS	48
7	Philip *et al.* [6]	2016	74	F	EGJ/mp/por	N(+)	45	Solitary	Surgery, IMRT	1
8	Kanazawa *et al.* [7]	2017	74	F	EGJ/ss/tub2	N(+)	4	Solitary	Surgery, SRS	60
9	Kostogloua *et al.* [8]	2019	71	F	L/ND/sig	ND	24	Solitary	Surgery	4
10	Lee WY *et al.* [9]	2023	65	M	U/ss/por	N(+)	24	Solitary	Surgery, CT	96
11	Our case	2024	59	F	U/ss/sig	N(+)	132	Two	Surgery, WBRT, CT	12

The locations of the stomach; U upper third, L lower third, EGJ: esophagogastric junction, ND: not described, se: exposition beyond the serosa, ss: subserosa invasion, mp: muscularis propria invasion, pap: papillary adenocarcinoma, tub1: well-differentiated adenocarcinoma, tub2: moderately differentiated adenocarcinoma, por: poorly differentiated adenocarcinoma, SRS: stereotactic radiosurgery, IMRT: intensity modulated radiotherapy, WBRT: whole brain radiotherapy, CT: chemotherapy.

Predicting prognosis is essential for determining treatment strategies for metastatic brain lesions. Sperduto *et al.* [10] proposed a Graded Prognostic Assessment (GPA). GPA scores predict prognosis according to age, Karnofsky Performance Status, number of central nervous system metastases, and the presence of extracranial metastasis. Moreover, the authors published a diagnosis-specific GPA that predicted the prognosis according to the primary cancer site [11]. Kasakura *et al.* [2] described the resection criteria for metastatic brain lesions. They suggested that to be suitable for surgery, metastatic brain tumours should satisfy some of the following requirements: (i) a single metastatic lesion in the brain, or if multiple, the lesions should be resectable by a single surgical intervention; (ii) a metastatic lesion located in an operable site; (iii) no metastasis to any other organs; and (iv) good general condition of the patient.

Radiotherapy is the preferred treatment after intracranial surgery. York *et al.* [2] reported that surgical resection with subsequent WBRT prolonged prognosis compared with WBRT or steroids alone. WBRT did not prolong the prognosis compared with steroid monotherapy. Along with WBRT, stereotactic radiosurgery (SRS) plays an important role in brain metastasis treatment. SRS is very useful because it can be applied to unresectable areas and can be performed repeatedly in the event of new lesions with few side effects. Moreover, SRS has a lower incidence of cognitive impairment than WBRT [12, 13].

In our case, two metastatic lesions were closely localized in the left cerebellum. Although the patient did not meet Kasakura’s criteria for metastasis to other organs, the condition was good. Therefore, we performed surgery for brain and adrenal gland metastases. After surgery, we administered WBRT and subsequent chemotherapy with S-1. We encountered an extremely rare case of late-onset metastatic recurrence of gastric cancer in a patient who survived for 1 year after the removal of metastatic lesions. There are no case reports in which recurrent brain and adrenal gland metastases have been resected.

Multidisciplinary treatments involving surgery, chemotherapy, and radiotherapy are required for patients with metastatic brain tumours. Although the metastatic recurrence of gastric cancer has a poor prognosis, multidisciplinary treatment, including surgical resection, may prolong the prognosis and provide a favourable quality of life. The possibility of resecting recurrent lesions, including brain metastases, should be considered based on the patient’s condition and tumour status.
